# Prevalence and associated factor of Campylobacter species among less than 5-year-old children in Ethiopia: a systematic review and meta-analysis

**DOI:** 10.1186/s40001-020-00474-7

**Published:** 2021-01-03

**Authors:** Kuma Diriba, Ephrem Awulachew, Asrat Anja

**Affiliations:** Department of Medical Laboratory Sciences, Health Science and Medical College, Dilla University, Dilla, Ethiopia

**Keywords:** Campylobacter, Prevalence, Under-five, Risk factor, Ethiopia

## Abstract

**Background:**

Despite the significant reductions in under-five mortality, campylobacteriosis has emerged as one of the most common causative agents of bacterial foodborne gastroenteritis in humans. We performed this systematic review and meta-analysis to estimate the pooled prevalence of Campylobacter species and associated risk factors among children less than 5 years of age in Ethiopia.

**Methods:**

A systematic search was conducted on PubMed, Web of Science, EMBASE, Google Scholar and the Cochrane Library. All identified observational studies reporting the prevalence and determinants of diarrhea among children under 5 years of age in Ethiopia were included. Two authors independently extracted data and analyzed them using STATA Version 13 statistical software. A random-effects model was computed to estimate the pooled prevalence and the associations between determinant factors and campylobacteriosis.

**Results:**

Out of 166 papers reviewed, 8 studies fulfilled the inclusion criteria and were included in the meta-analysis. The pooled prevalence of Campylobacter species among children under 5 years of age in Ethiopia was 10% (95% CI: 7, 13). Contact with domestic animals (OR: 3.2, 95% CI: 2.0, 5.1), illiterate mothers (OR: 2.1, 95% CI: 1.1, 3.8), consumption of animal products (OR: 1.7, 95% CI: 0.7, 4.5), and status of mothers’ personal hygiene (OR: 1.1, 95% CI: 0.7, 1.8) were significantly associated with the prevalence of Campylobacter species.

**Conclusion:**

In our study, Campylobacter species among children under 5 years of age in Ethiopia were significantly high. Contact with domestic animals, illiterate mothers and consumption of animal products were significantly associated with prevalence of Campylobacter species

## Background

Campylobacter species are small Gram-negative, thermotolerant, helical microorganisms with a unique ‘darting’ motility with a single polar flagellum and grow in microaerobic conditions within the range of 30–42 °C [1, 2]. The Campylobacter genus has increased drastically; and presently incorporates 17 species and 6 subspecies, many of which might be associated with human disease [3]. Most human campylobacteriosis results from *Campylobacter jejuni* and *Campylobacter coli* [4]. Both species are zoonotic pathogens with wide host ranges including livestock (cattle, sheep, pigs and poultry) and wild animals [5, 6].

*Campylobacter* has become one of the most common causative agents of both diarrheal and systemic diseases. The incidence of human *Campylobacter* infections is increasing worldwide [7]. Currently, it is the leading cause of bacterial gastroenteritis [8, 9]. The infection is transmitted through the oral route from food, drink, or contact with infected animals or animal products [10]. Animals, including poultry, beef, pork, sheep and goats, are natural reservoir hosts for *Campylobacter* species [11–13]. Recent studies report a wide range (5–49%) of *Campylobacter* prevalence in healthy sheep and goats. Human exposure can come through direct contact with animal food [14–18]. Flies play a crucial role in the transmission of *Campylobacter* species from contaminated sources to broiler chickens [19].

Infectious diarrheal diseases are of remarkable concern, as they are responsible for more than 95 million foodborne illnesses and greater than 21,000 deaths [20–24]. Acute infection by Campylobacter can cause serious long-term consequences, including peripheral neuropathies, Guillain–Barre syndrome [25] and Miller Fisher syndrome [1], and functional bowel diseases which include irritable bowel syndrome [20]. Diarrhea is highly prevalent in sub-Saharan Africa, which incorporates Ethiopia, which results in the highest rates of child mortality [26, 27]. Campylobacter infections are commonly mild but may be fatal among very young children, elderly and immune-suppressed individuals and often occur more frequently per year than Salmonella species, Shigella species [28].

The growing rate of human infections because of antimicrobial resistance strains of Campylobacter makes clinical management more difficult by prolonging the infection and compromising the treatment. This can have a probably an extreme impact on food safety in both animal and human health. The scenario appears to deteriorate more hastily in growing countries in which there is giant and uncontrolled use of antibiotics [7, 28]. Data on Campylobacter species among children under 5 years of age in Ethiopia are limited and are not currently available in aggregate form. Therefore, we conducted this systematic review and meta-analysis to determine the pooled prevalence and determinants of human campylobacteriosis among children under 5 years of age using available studies in Ethiopia.

## Methods

### Study design

A systematic review and meta-analysis were conducted to estimate the prevalence and determinant of Campylobacter species in under-five children in Ethiopia following the methodological framework suggested by Arksey and O’Malley [29].

### Search strategies

All relevant articles were searched without date limits using the following databases: PubMed, Web Science, Embase, Google Scholar, Cochrane Library and Science Direct according to the Preferred Reporting Items for Systematic Reviews and Meta-analysis (PRISMA) [30]. All searches were limited to articles written in English given that such language restriction does not alter the outcome of the systematic reviews and meta-analysis [31]. The gray literature of observational studies was searched through the review of reference lists and input of content experts. The literature search was conducted from November 1/2019 to December 10/2019. All papers published until the end of 2017 and fulfilling inclusion criteria were considered. The search used the following keywords “campylobacter”, “prevalence”, “under-five”, “children”, “risk factor”, “associated factors” and “Ethiopia”. We searched all terms with the help of Boolean operators such as “AND” or “OR”.

### Eligibility criteria

Studies conducted only in Ethiopia and involving only humans were included in this study. Publication condition: only published articles were included. Study design: all observational study designs reporting the prevalence of Campylobacter species in humans were eligible for this review. Language: only articles reported in English language were considered. Exclusion criteria: articles that were not fully accessible, after email contact with the primary authors and duplicate publications of the same study, were excluded.

### Assessment of study quality

Studies selected for inclusion were assessed for methodological quality by all authors independently using the standard critical appraisal instruments of the Joanna Briggs Institute Meta-Analysis of Statistics Assessment for Review Instrument (JBI-MAStARI) [32]. Disagreements were resolved by consensus.

### Outcome measure

The primary outcome variable of this study was the prevalence of Campylobacter species, while secondary outcomes were all associated risk factors identified as listed below: history of contact with domestic animals, residence of study participants, history of consumption of animal products, usage of clean water**,** educational background study participants, status of personal hygiene and status of latrine usage of study participants.

### Data extraction

Data were extracted using a standardized data extraction format, adapted from the Joanna Briggs Institute (JBI), by three authors (Kuma Diriba Asrat Anja and Ephrem Awulachew) independently extracting all necessary data. Then the extracted data were merged for systematic analysis. Any disagreements during the data extraction were resolved through discussion and consensus. The main outcomes extracted from the study were: primary author, publication year, study method, study area, sample size and cases. Data on associated risk factors were also extracted by the authors.

### Statistical analysis

Following data extraction, systematic review and meta-analysis were carried out using R software version 3.6.1 and STATA statistical software (version 13) with user-contributed commands for meta-analyses: metaprop, metan, metainf, metabias, and metareg [33]. The effect sizes and SEs of the studies were pooled using a random-effects model to calculate the pooled prevalence of Campylobacter species in less than 5-year-old children in Ethiopia. A meta-analysis was also planned to assess the association of various associated factors, such as history of contact with domestic animal, residence of study participant, history of consumption of animal product, usage of clean water**,** educational background study participant, status of personal hygiene and status of latrine usage of study participant.

### Risk of bias

Three authors (KD, AA and EA) independently assessed the risk of bias for each original study using the 10 criteria tool of Hoy 2012, which addresses internal and external validity [34]. The tool mainly included (1) representation of the population; (2) sampling frame; (3) methods of participants’ selection; (4) non-response bias; (5) data collection directly from subjects; (6) acceptability of case definition; (7) reliability and validity of study tools;(8) mode of data collection; (9) length of prevalence period, and (10) appropriateness of numerator and denominator. Each item was classified as either low or high risk of bias. Finally, the overall score of risk of bias was then categorized into low (2), moderate (3–4), and high (> 5) out of 10 and almost all of the original studies fall under low risk of bias.

The standard error for each original study was calculated using the binomial distribution formula. Evidence for statistical heterogeneity among reported prevalence was using the Cochrane Q-test and I^2^ statics [35]. The pooled proportion was estimated by using the back-transform of the weighted mean of the transformed proportions for both the fixed-effects model and the random-effects model [36]. A significance level of *P* < 0.10 and *I*^2^ > 50% was interpreted as evidence of heterogeneity [37]. A potential source of heterogeneity was investigated by subgroup analysis and meta-regression analysis [38]. Where statistical pooling was not possible, the findings were presented in a narrative form including tables and figures to aid in data presentation where appropriate.

### Sensitivity analysis

Sensitivity analyses were conducted to weigh up the relative influence of each individual study on the pooled effect size using a user-written function, metainf. The presence of publication bias was assessed informally by visual inspection of funnel plots [39]. Point prevalence, as well as 95% confidence intervals, was presented in the forest plot format.

## Results

### Study selection

**Fig. 1:**
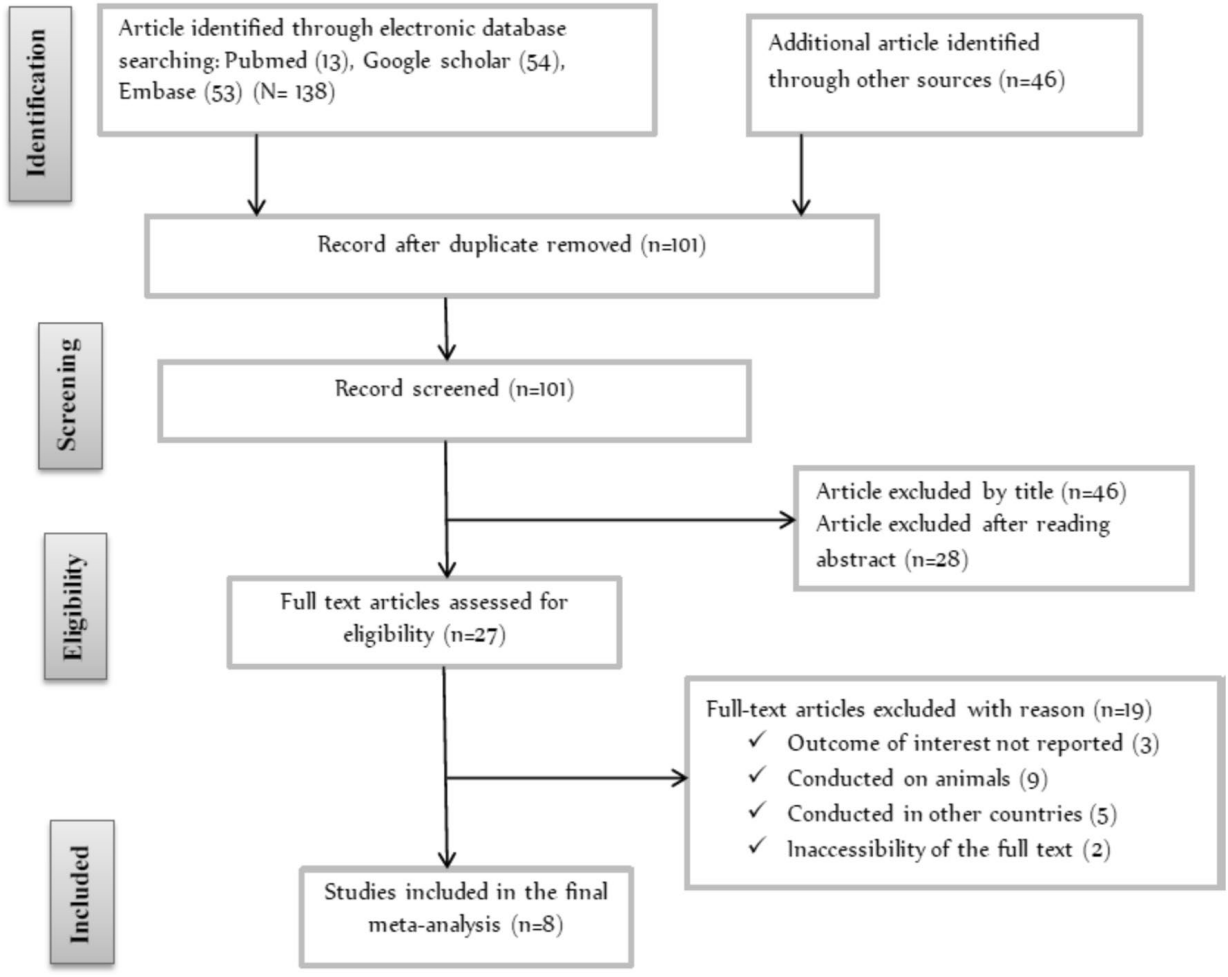
Flowchart of study selection for systematic review and meta-analysis of the prevalence and associated factors of Campylobacter species among less than 5-year-old children in Ethiopia

A database search identified a total of 166 articles reporting the prevalence of Campylobacter species among children less than 5 years of age. From these initial articles, 65 articles were excluded due to duplication/repeated. From the remaining 101 articles, 74 articles were excluded after review of their titles and abstracts confirmed non-relevance to this review, 27 articles were assessed with respect to their eligibility for inclusion, which resulted in the further exclusion of 19 articles primarily due to the study done on animals [40–53], and 8 studies were included in the final systematic review and meta-analysis (Fig. 1).

### Description of included studies

In this review, 8 papers published between 1997 and 2017 and reported the prevalence and associated factors of Campylobacter species among children younger than 5 years of age were included in this study. In this systematic review and meta-analysis, 2250 study participants were included to assess the pooled prevalence of Campylobacter species among children less than 5 years of age. A total sample size of the included articles ranged from 153 to 630. The lowest [54] and the highest [55] prevalence (3.5%) of Campylobacter species were reported from the same site, Jimma town, Oromia region, with prevalence of 3.5% and 16.7%, respectively. Of the included studies, three studies were from Amhara [56–58], two from SNNPR [59, 60], two from Oromia [54, 55] and one from Addis Ababa [61] (Table 1).

**Table 1:** Descriptive summary of 8 studies included in the meta-analysis of the prevalence and associated risk factors of campylobacteriosis among less than 5-year-old children in Ethiopia, 2019

First authors	Publication year	Study method	Study area	Region	Sample size	Cases	Prevalence with 95% CI
Mulatu [59]	2014	Cross-sectional	Hawassa town	SNNPR	158	20	13 (8–19)
Tafa [55]	2014	Cross-sectional	Jimma town	Oromia	227	38	17 (12–22)
Lengerh [56]	2013	Cross-sectional	Gondar town	Amhara	285	44	15 (11–20)
Kebede [60]	2017	Cross-sectional	Hawassa town	SNNPR	215	13	6 (3–10)
Asrat [61]	1997	Case control	Addis Ababa city	Addis Ababa	630	66	10 (8–13)
Ewunatu [57]	2010	Cross-sectional	Bahir Dar town	Amhara	210	16	8 (4–12)
Awole [54]	2002	Cross-sectional	Jimma town	Oromia	372	13	3 (2–6)
Mitike [58]	2000	Cross-sectional	Dembia district	Amhara	153	16	10 (7–16)

### Risk of bias

The risk of bias tool [34] was used to assess the risk of bias for the included studies and almost greater than 80% of the studies had a low risk of bias. The sample selection and temperature during transport and the amount of any individual sample tested were specified in some of the studies. *Campylobacter* specific liquid and solid media were used in the majority of the studies. *Campylobacter* was incubated microaerophilically or in a candle jar in most of the studies.

### Prevalence of Campylobacter species among less than 5 years in Ethiopia

**Fig. 2:**
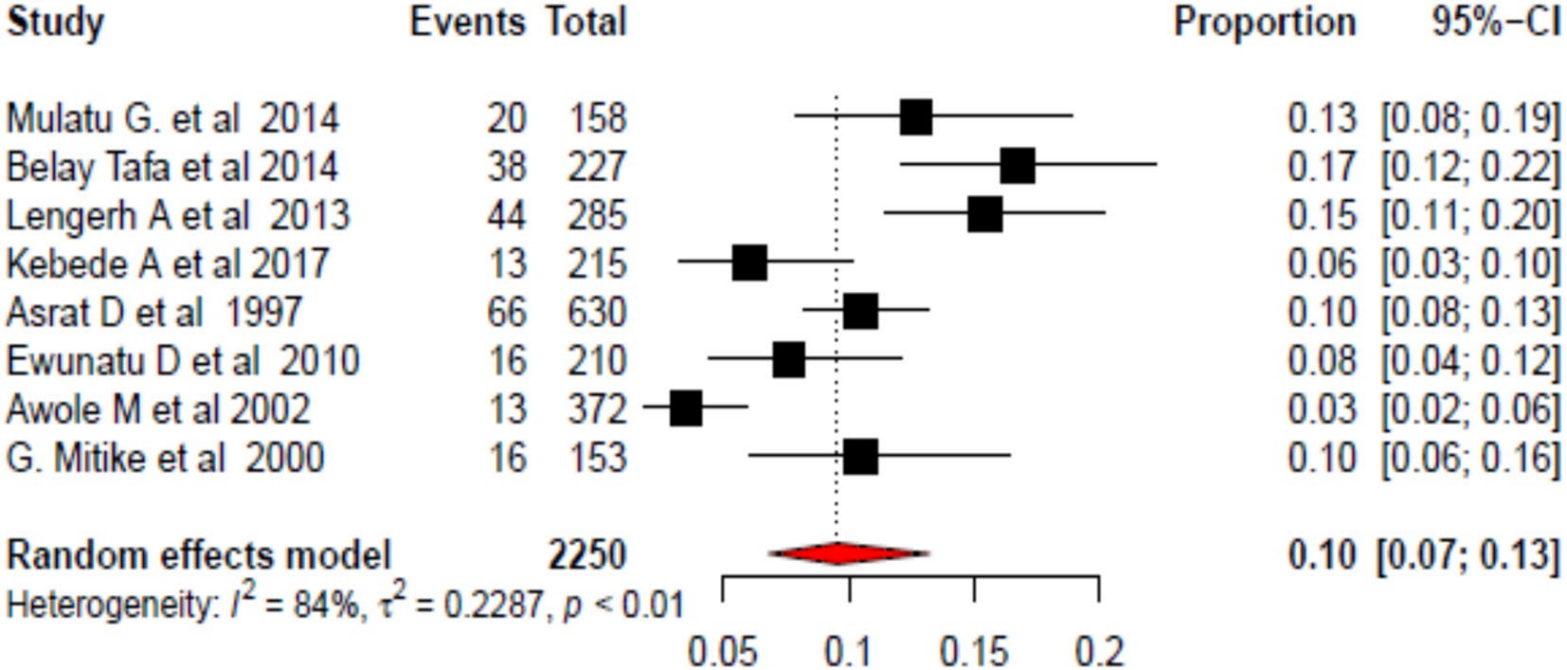
Forest plot of the pooled prevalence of Campylobacter species among under-five children in Ethiopia

The pooled prevalence of Campylobacter species in children less than 5 years of age in Ethiopia was 10% (95% CI: 7–13). Due to the presence of high heterogeneity (*I*^2^ = 84, *p* < 0.01), a random effect meta-analysis model was explored to assess the pooled prevalence of Campylobacter species in children less than 5 years of age in Ethiopia (Fig. 2).

**Fig. 3:**
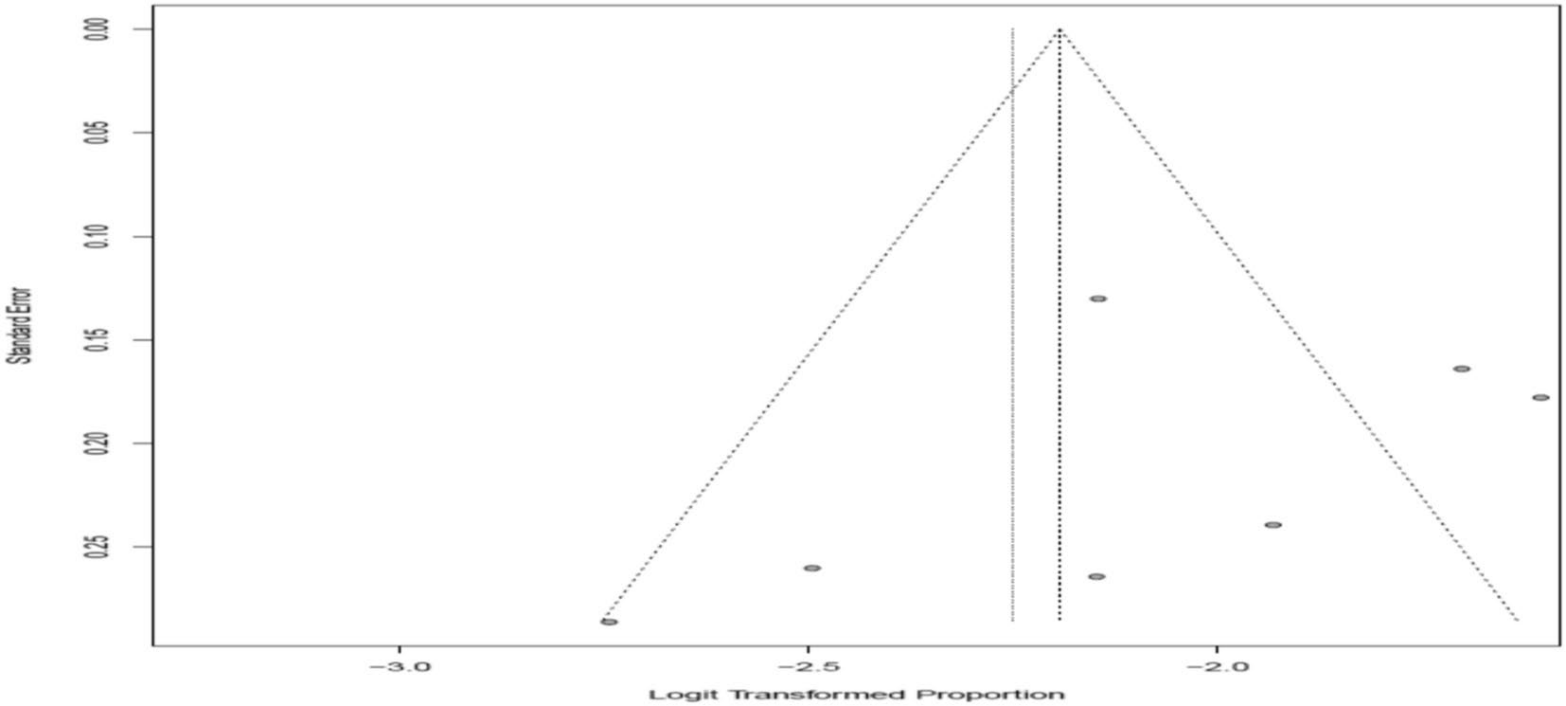
Funnel plot with 95% confidence limits of the pooled prevalence of Campylobacter species among under-five children in Ethiopia

For identification of the source of heterogeneity, we assessed the year when the study was published, the place where the study was done and the sample size using univariate meta-regression models. However, all differences were not statistically significant. The funnel plot showed some irregular distribution of articles. In our systematic review and meta-analysis, the highest prevalence, 17% (95% CI: 12, 22) [55], and the lowest prevalence, 3% (95% CI: 2, 6) [54] were reported from the same study area which may be due to the study period and method and media used (Table 2, Fig. 3).

**Table 2:** Campylobacter species prevalence among children less than 5 years old and assessment of source of heterogeneity in Ethiopia, 2019

Variable	Coefficient	*P* value
Publication year	0.23	0.27
Sample size	0.23	0.02
*Region*		
Oromia	0.70	0.01
Amhara	0.07	0.03
SNNPR	0.09	0.03
Addis Ababa	NA	NA

### Subgroup analysis

In the present study, subgroup analysis was performed based on the study area. Based on subgroup analysis, the Amhara region was the leading study area followed by Addis Ababa city and SNNPR, with a prevalence of 11% (95% CI: 8, 16), 10% (95% CI: 8, 13) and 9% (95% CI: 5, 15), respectively (Table 3).

**Table 3:** Prevalence of Campylobacter species among children less than 5 years old by subgrouping in Ethiopia, 2019 (*n* = 8)

Variable	Characteristics	Included study	Sample size	Prevalence with (95% CI)
Region	Addis Ababa	1	630	10 (0.08–0.13)
	Oromia	2	373	8 (0.01–0.22)
	Amhara	3	599	11 (0.08–0.16)
	SNNPR	2	373	9 (0.05–0.15)

### Risk factor associated with campylobacteriosis in Ethiopia

#### Association between contact with domestic animals and campylobacteriosis

**Fig. 4:**
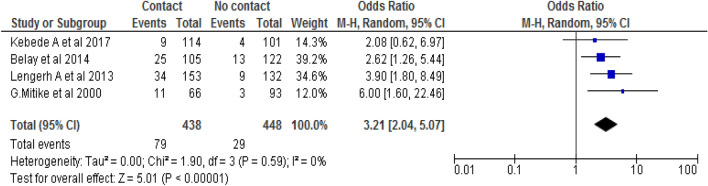
The pooled odds ratio of the association between campylobacteriosis and domestic animals in Ethiopia

In the current study, the association between contact with domestic animals like cat, dog, hen and pigeon and campylobacteriosis was assessed by using four studies [55, 56, 58, 60]. The association showed that the occurrence of campylobacteriosis was significantly associated with contact with domestic animals. Based on this, the likelihood of campylobacteriosis occurrence was 3.2 times higher among children who contact domestic animals than among those children who do not contact domestic animal counterparts (OR: 3.2, 95% CI: 1.8, 8.5). No heterogeneity was observed during this analysis (*I*^2^ = 0% and *p* < 0.0001). Therefore, we explored random effect meta-analysis model to assess the association (Fig. 4).

#### The association between the educational background of the mother and campylobacteriosis

**Fig. 5:**
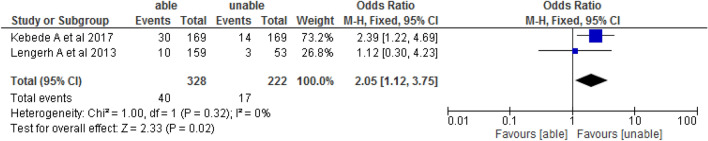
The pooled odds ratio of the association between mother educational background and prevalence of Campylobacter species in Ethiopia, 2019

In this study, we also assessed the association between the educational background of the mother and campylobacteriosis by using two studies [56, 60]. The association showed that the presence of campylobacteriosis was significantly associated with mothers’ educational status that have less knowledge and information about the appropriate application of personal hygiene for themselves and to their children. Based on this, the likelihood of campylobacteriosis presence was 2.1 times higher among children whose mothers were illiterate compared to the educated mothers (OR: 2.1, 95% CI: 1.1, 3.8) (Fig. 5).

#### Association between consumption of animal product and campylobacteriosis

**Fig. 6:**
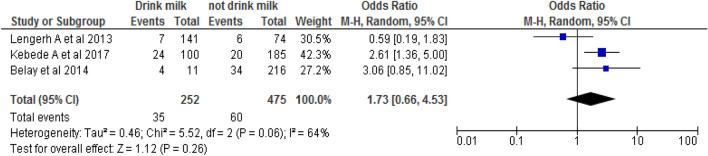
The pooled odds ratio of the association between the status of drinking milk and the prevalence of Campylobacter species in Ethiopia, 2019

Three studies [55, 56, 60] were used to assess the association between the consumption of animal products and campylobacteriosis. Patients who drink milk and milk products were significantly associated with the presence of campylobacteriosis. Based on this, the likelihood of Campylobacter species occurrence was 1.7 times higher among children who drink milk than among children who do not drink milk (OR: 1.7, 95% CI: 0.7, 4.5) (Fig. 6).

In this systematic review and meta-analysis, we also assessed the association between personal hygiene, use of clean water, residence of study participants, presence of latrines and prevalence of Campylobacter species. None of them had an association with the prevalence of Campylobacter species in this study (Table 4).

**Table 4:** The pooled odds ratio of the association between the stated risk factor and prevalence of Campylobacter species among under-five children in Ethiopia, 2019 (*n* = 8)

Variables	Studies included	Participants	OR (95% CI)
Contact with domestic	4	886	3.21 (2.04, 5.07)
Caregiver able to read and write	2	550	2.05 (1.12, 3.75)
Consumption of animal product	3	727	1.73 (0.66, 4.53)
Hand wash of caregiver	2	697	1.10 (0.67, 1.83)
Residence	2	500	0.76 (0.43, 1.35)
Latrine use	2	500	0.39 (0.20, 0.75)
Protected water used	3	727	0.44 (0.23, 0.830

## Discussion

Data on Campylobacter species among children less than 5 years of age in Ethiopia are limited and are not currently available in aggregate form. We conducted a systematic review and meta-analysis to provide the pooled prevalence of Campylobacter species and associated risk factors. Campylobacter is one of four key global causes of diarrheal diseases [62]. It is considered to be the most common bacterial cause of human gastroenteritis in the world with an estimated 400 million cases per year [8, 62]. The information from this study might be used by policy makers in the prevention and control of infection.

In the present study, the pooled prevalence of Campylobacter species in children less than 5 years of age indicated that almost one in ten (10%) suffered from with campylobacteriosis. The finding of our study is consistent with studies conducted in Uganda [63], Zimbabwe [64], Egypt [65] and Madagascar [66] with prevalences of 9.3, 9.3, 9 and 9.7%, respectively. However, this finding is lower than those of studies conducted in South Africa [67] and Tanzania [68] with prevalence of 24.9 and 18%, respectively. Similarly, the result of this meta-analysis is much higher than the study conducted in Mozambique [69] 1.7%. The possible explanation for this differences might be methodological variation, socio-demographics and cultural difference, which have a great impact on child feeding.

In the current study, the highest prevalence of Campylobacter species was observed in the Amhara region [56–58], with a prevalence of 11% followed by Addis Ababa city [70] 10%, whereas the lowest prevalence was observed in Oromia [54, 55] and SNNPR [60], with prevalences of 8 and 9%, respectively. The results of this study showed that the prevalence of Campylobacter species throughout the region is almost the same. However, the small variation might be due to the differences in socio-demographic, environmental and sociocultural factors. Moreover, quality of life might be affected by a lack of education and poverty, which may be directly associated with the occurrence of campylobacteriosis among children less than 5 years of age.

In the present study, contact with domestic animals, uneducated mothers and drinking of milk before boiling were significantly associated with the prevalence of Campylobacter species. The likelihood of campylobacteriosis occurrence was 3.2 times higher among children who contact domestic animals than among those children who do not contact domestic animals. Our study result is similar to studies performed in Colorado [71], Kenya [72], Egypt [9, 65] and elsewhere [56], which showed that the prevalence of campylobacteriosis was significantly higher in children who contact domestic animals.

The results of this meta-analysis showed that campylobacteriosis was higher among children whose mothers had no formal education. This is in line with a study conducted in England [73], but a study conducted in New Zealand [74] showed high educational attainment, and home ownership greater than 50% was associated with increased an incidence of Campylobacter infection. This result may be associated with these literate mothers having better knowledge and information about the appropriate application of personal hygiene for themselves and to their children, and they also have adequate information on the clinical features of diarrhea during its occurrence. Educated mothers had better awareness than uneducated mothers, and they know about the mechanism of transmission and prevention and control of diarrhea due to campylobacteriosis.

Finally, children who drink milk before boiling and those who eat raw meat were more vulnerable to campylobacteriosis than those who do not drink milk before boiling or eating raw meat. Based on this, the likelihood of Campylobacter species occurrence was 1.7 times higher among children who drink milk than among those who do not drink milk before boiling. This finding is consistent with a study conducted in different areas of Ethiopia [41, 55, 56, 60]. The consumption of animal products may alter the ecology and epidemiology of Campylobacter in the environment and people, which may drive the emergence of new epidemiological patterns of disease.

## Limitations of the study

The collected article for this study was limited to the English language. The study method (most were cross-sectional) can affect the outcome variable by other confounding variables. Small sample size could affect the estimated pooled prevalence of Campylobacter species. Therefore, this meta-analysis represented only studies reported from a limited study area, which may reflect underrepresentation due to the limited number of studies included.

## Conclusion

In this study, Campylobacter species among children less than 5 years of age in Ethiopia was significantly high. Regarding the associated risk factors, contact with domestic animals, illiterate mothers, consumption of animal products, and mothers who do not use proper personal hygiene were found to be significantly associated with the prevalence of Campylobacter species. Therefore, based on our findings, we recommend emphasis shall be given on health education about the protection of contact with domestic animals, cooking of animal products before consumption, personal hygiene and proper disposal of wastes including excreta in integration with the existing national health extension program.

## Data Availability

The datasets used and/or analyzed during the current study are available from the corresponding author upon reasonable request.

## Abbreviations

CI: Confidence interval
JBI: Joanna Briggs Institute
N: Number
OR: Odds ratio
SNNPR: Southern Nation National Peoples Region
High heterogeneity: Variability among studies in a systematic review is greater than 50% (*I*^2^ > 50%)
Zero heterogeneity: No variability among studies in a systematic review (*I*^2^ = 0%)

## References

[1] Tumbarski Y (2019). Epidemiology and Prevalence of Campylobacter Infections in the European Union and Bulgaria between 2010 and 2017 (A Review). Bulgarian J Vet Med.

[2] Qu M, Zhang M, Zhang X, Jia L, Xu J, Chu Y (2019). Molecular and epidemiological analysis of a Campylobacter jejuni outbreak in China, 2018. J Infect Dev Ctries.

[3] Soofi SB, Habib MA, Von Seidlein L, Khan MJ, Muhammad S, Bhutto N (2011). A comparison of disease caused by Shigella and Campylobacter species: 24 months community based surveillance in 4 slums of Karachi, Pakistan. J Infect Public Health.

[4] Kaur T, Singh J, Huffman MA, Petrželková KJ, Taylor NS, Xu S (2011). Campylobacter troglodytis sp. nov., isolated from feces of human-habituated wild chimpanzees (Pan troglodytes schweinfurthii) in Tanzania. Appl Environ Microbiol..

[5] de Jong M. A One Health approach towards artificial insemination in cattle in Tanga, Tanzania 2019.

[6] Kaakoush NO, Castaño-Rodríguez N, Mitchell HM, Man SM (2015). Global epidemiology of Campylobacter infection. Clin Microbiol Rev.

[7] Altekruse SF, Stern NJ, Fields PI, Swerdlow DL (1999). Campylobacter jejuni—an emerging foodborne pathogen. Emerg Infect Dis.

[8] Dabboussi F, Alam S, Mallat H, Hlais S, Hamze M (2012). Preliminary study on the prevalence of Campylobacter in childhood diarrhoea in north Lebanon. East Mediterr Health J.

[9] Coker AO, Isokpehi RD, Thomas BN, Amisu KO, Obi CL (2002). Human campylobacteriosis in developing countries1. Emerg Infect Dis.

[10] Zeigler M, Claar C, Rice D, Davis J, Frazier T, Turner A (2014). Outbreak of campylobacteriosis associated with a long-distance obstacle adventure race—Nevada, October 2012. MMWR Morb Mortal Wkly Rep.

[11] Hald B, Skov MN, Nielsen EM, Rahbek C, Madsen JJ, Wainø M (2015). Campylobacter jejuni and Campylobacter coli in wild birds on Danish livestock farms. Acta Vet Scand.

[12] Josefsen MH, Bhunia AK, Engvall EO, Fachmann MS, Hoorfar J (2015). Monitoring Campylobacter in the poultry production chain—From culture to genes and beyond. J Microbiol Methods.

[13] Skarp C, Hänninen M-L, Rautelin H (2016). Campylobacteriosis: the role of poultry meat. Clin Microbiol Infect.

[14] Garcia A, Steele W, Taylor D (2010). Prevalence and carcass contamination with Campylobacter in sheep sent for slaughter in Scotland. J Food Saf.

[15] Rahimi E, Kazemeini HR, Safaei S, Allahbakhshi K, Momeni M, Riahi M (2010). Detection and identification of Campylobacter spp. from retail raw chicken, turkey, sheep and goat meat in Ahvaz, Iran. Afr J Microbiol Res.

[16] Salihu M, Junaidu A, Oboegbulem S, Egwu G (2009). Prevalence and biotypes of Campylobacter species isolated from sheep in Sokoto State, Nigeria. Int J Animal Vet Adv.

[17] Lévesque S, Frost E, Arbeit RD, Michaud S (2008). Multilocus sequence typing of Campylobacter jejuni isolates from humans, chickens, raw milk, and environmental water in Quebec, Canada. J Clin Microbiol.

[18] Rahimi E, Ameri M, Kazemeini HR (2010). Prevalence and antimicrobial resistance of Campylobacter species isolated from raw camel, beef, lamb, and goat meat in Iran. Foodborne Pathogens Dis.

[19] Jonsson ME, Chriél M, Norström M, Hofshagen M (2012). Effect of climate and farm environment on Campylobacter spp. colonisation in Norwegian broiler flocks. Prev Vet Med.

[20] Kaba M, Ayele F (2000). Ethnographic study of diarrhoeal diseases among under-five children in Mana district, Jimma Zone, Southwest Ethiopia. Ethiop J Health Dev.

[21] Meseret E (1994). Analysis of pediatric admission to Jimma Hospital pediatric ward: a three year retrospective study. Bull JIHS.

[22] Jafari F, Shokrzadeh L, Hamidian M, Salmanzadeh-Ahrabi S, Zali MR (2008). Acute diarrhea due to enteropathogenic bacteria in patients at hospitals in Tehran. Jpn J Infect Dis.

[23] Gill CJ, Thea DM, Hibberd P (2017). Diarrhoeal disease trends in the GBD 2015 study: optimism tempered by scepticism. Lancet Infect Dis.

[24] Liu L, Oza S, Hogan D, Perin J, Rudan I, Lawn JE (2015). Global, regional, and national causes of child mortality in 2000–13, with projections to inform post-2015 priorities: an updated systematic analysis. Lancet.

[25] Esan OB, Pearce M, van Hecke O, Roberts N, Collins DR, Violato M (2017). Factors associated with sequelae of Campylobacter and non-typhoidal Salmonella infections: a systematic review. EBioMedicine.

[26] Ramana J (2012). dbDiarrhea: The database of pathogen proteins and vaccine antigens from diarrheal pathogens. Infect Genet Evol.

[27] Chan M, Lake A (2012). WHO/UNICEF on ending preventable child deaths. Lancet.

[28] Acheson D, Allos BM (2001). Campylobacter jejuni infections: update on emerging issues and trends. Clin Infect Dis.

[29] Arksey H, O'Malley L (2005). Scoping studies: towards a methodological framework. Int J Soc Res Methodol.

[30] Liberati A, Altman DG, Tetzlaff J, Mulrow C, Gøtzsche PC, Ioannidis JP (2009). The PRISMA statement for reporting systematic reviews and meta-analyses of studies that evaluate health care interventions: explanation and elaboration. PLoS medicine.

[31] Moher D, Pham B, Lawson M, Klassen T (2003). The inclusion of reports of randomised trials published in languages other than English in systematic reviews. Health Technol Assess.

[32] Armstrong R, Waters E, Jackson N (2007). Systematic reviews of health promotion and public health interventions.

[33] Cheng Z, Lu Y, Cao Q, Qin L, Pan Z, Yan F (2019). Clinical features and chest CT manifestations of coronavirus disease 2019 (COVID-19) in a single-center study in Shanghai, China. Am J Roentgenol.

[34] Hoy D, Brooks P, Woolf A, Blyth F, March L, Bain C (2012). Assessing risk of bias in prevalence studies: modification of an existing tool and evidence of interrater agreement. J Clin Epidemiol.

[35] Rücker G, Schwarzer G, Carpenter JR, Schumacher M (2008). Undue reliance on I 2 in assessing heterogeneity may mislead. BMC Med Res Methodol.

[36] Nyaga VN, Arbyn M, Aerts M (2014). Metaprop: a Stata command to perform meta-analysis of binomial data. Arch Public Health.

[37] Thompson SG, Sharp SJ (1999). Explaining heterogeneity in meta-analysis: a comparison of methods. Stat Med.

[38] Cochran WG (1950). The comparison of percentages in matched samples. Biometrika.

[39] Egger M, Smith GD, Schneider M, Minder C (1997). Bias in meta-analysis detected by a simple, graphical test. BMJ.

[40] Desta AH (2016). Community Based Intervention for Zoonotic Diseases Prevention and Control in Ethiopian Pastoral Areas. J Pharm Altern Med.

[41] Kassa T, Gebre-selassie S, Asrat D (2005). The prevalence of thermotolerant Campylobacter species in food animals in Jimma Zone, southwest Ethiopia. Ethiop J Health Dev.

[42] Woldemariam T, Asrat D, Zewde G (2009). Prevalence of thermophilic Campylobacter species in carcasses from sheep and goats in an abattoir in Debre Zeit area, Ethiopia. Ethiop J Health Dev.

[43] Kassa T, Gebre-Selassie S, Asrat D (2007). Antimicrobial susceptibility patterns of thermotolerant Campylobacter strains isolated from food animals in Ethiopia. Vet Microbiol.

[44] Chanyalew Y, Asrat D, Amavisit P, Loongyai W (2013). Prevalence and antimicrobial susceptibility of thermophilic Campylobacter isolated from sheep at Debre Birhan, North-Shoa, Ethiopia. Kasetsart J.

[45] Hailemariam S, Feleke A, Szonyi B, Fries R, Baumann M, Grace D. Prevalence and antimicrobial susceptibility pattern of thermophilic Campylobacter spp. isolated from ovine carcasses and faeces in Ethiopia. International Livestock Research Institute: 2015. https://hdl.handle.net/10568/68018.

[46] Desta AH (2016). One health: an integrated approach for disease prevention and control in pastoral areas of Ethiopia. J Health Med Nur.

[47] Mekkonen Y, Brena MC, Christley R, Bettridge JM, Collins M, Dessie T, Tessema TS. Detection of Campylobacter carriage rate in different poultry production systems in Ethiopia. Society of Veterinary Epidemiology and Preventive Medicine, 2013. https://hdl.handle.net/10568/64989.

[48] Ejo M, Garedew L, Alebachew Z, Worku W (2016). Prevalence and antimicrobial resistance of Salmonella isolated from animal-origin food items in Gondar, Ethiopia. BioMed Res Int.

[49] Haileselassie M, Taddele H, Adhana K, Kalayou S (2013). Food safety knowledge and practices of abattoir and butchery shops and the microbial profile of meat in Mekelle City, Ethiopia. Asian Pac J Trop Biomed.

[50] Brena M, Mekonnen Y, Bettridge J, Williams N, Wigley P, Tessema TS (2016). Changing risk of environmental Campylobacter exposure with emerging poultry production systems in Ethiopia. Epidemiol Infect.

[51] Tegegne HA, Berhanu A, Getachew Y, Serda B, Nölkes D, Tilahun S (2019). Microbiological safety and hygienic quality of camel meat at abattoir and retail houses in Jigjiga city, Ethiopia. J Infect Dev Ctries.

[52] Kebede T, Afera B, Taddele H, Bsrat A (2014). Assessment of bacteriological quality of sold meat in the butcher shops of Adigrat.

[53] Mekuria A, Beyene T (2014). Zoonotic bacterial pathogens isolated from food of bovine in selected Woredas of Tigray. Ethiopia World Appl Sci J.

[54] Awole M, Gebre-Selassie S, Kassa T, Kibru G (2002). Isolation of potential bacterial pathogens from the stool of HIV-infected and HIV-non-infected patients and their antimicrobial susceptibility patterns in Jimma Hospital, south west Ethiopia. Ethiop Med J.

[55] Tafa B, Sewunet T, Tassew H, Asrat D (2014). Isolation and antimicrobial susceptibility patterns of Campylobacter species among diarrheic children at Jimma, Ethiopia. Int J Bacteriol.

[56] Lengerh A, Moges F, Unakal C, Anagaw B (2013). Prevalence, associated risk factors and antimicrobial susceptibility pattern of Campylobacter species among under five diarrheic children at Gondar University Hospital, Northwest Ethiopia. BMC Pediatr.

[57] Ewnetu D, Mihret A (2010). Prevalence and antimicrobial resistance of Campylobacter isolates from humans and chickens in Bahir Dar. Ethiopia Foodborne Pathogens Dis.

[58] Mitike G, Kassu A, Genetu A, Nigussie D. Campylobacter enteritis among children in Dembia district, northwest Ethiopia. East Afr Med J. 2000;77(12).

[59] Mulatu G, Beyene G, Zeynudin A (2014). Prevalence of Shigella, Salmonella and Campylobacter species and their susceptibility patters among under five children with diarrhea in Hawassa Town, South Ethiopia. Ethiop J Health Sci.

[60] Kebede A, Aragie S, Shimelis T (2017). The common enteric bacterial pathogens and their antimicrobial susceptibility pattern among HIV-infected individuals attending the antiretroviral therapy clinic of Hawassa University Hospital, Southern Ethiopia. Antimicrob Resist Infect Control.

[61] Asrat D, Hathaway A, Sjögren E, Ekwall E, Kaijser B (1997). The serotype distribution of Campylobacter jejuni and C. coli isolated from patients with diarrhoea and controls at Tikur Anbassa Hospital, Addis Ababa, Ethiopia. Epidemiol Infect.

[62] Oliver SP (2019). Foodborne Pathogens and Disease Special Issue on the National and International PulseNet Network. Foodborne Pathogens Dis.

[63] Mshana S, Joloba M, Kakooza A, Kaddu-Mulindwa D (2009). Campylobacter spp among Children with acute diarrhea attending Mulago hospital in Kampala-Uganda. Afr Health Sci.

[64] Karikari AB, Obiri-Danso K, Frimpong EH, Krogfelt KA (2017). Antibiotic resistance in Campylobacter isolated from patients with gastroenteritis in a teaching hospital in Ghana. Open J Med Microbiol.

[65] Rao MR, Naficy AB, Savarino SJ, Abu-Elyazeed R, Wierzba TF, Peruski LF (2001). Pathogenicity and convalescent excretion of Campylobacter in rural Egyptian children. Am J Epidemiol.

[66] Randremanana R, Randrianirina F, Gousseff M, Dubois N, Razafindratsimandresy R, Hariniana ER (2012). Case-control study of the etiology of infant diarrheal disease in 14 districts in Madagascar. PLoS ONE.

[67] Samie A, Ramalivhana J, Igumbor E, Obi C (2007). Prevalence, haemolytic and haemagglutination activities and antibiotic susceptibility profiles of Campylobacter spp. isolated from human diarrhoeal stools in Vhembe District, South Africa. J Health Popul Nutr.

[68] Lindblom G-B, Åhrén C, Changalucha J, Gabone R, Kaijser B, Nilsson L-Å (1995). Campylobacter jejuni/coli and Enterotoxigenic Escherichia coli (ETEC) in Faeces from Children and Adults in Tanzania. Scand J Infect Dis.

[69] Mandomando IM, Macete EV, Ruiz J, Sanz S, Abacassamo F, Valles X (2007). Etiology of diarrhea in children younger than 5 years of age admitted in a rural hospital of southern Mozambique. Am J Trop Med Hyg.

[70] Asrat D. Shigella and Salmonella serogroups and their antibiotic susceptibility patterns in Ethiopia. 2008. 19166157

[71] Hopkins RS, Olmsted R, Istre GR (1984). Endemic Campylobacter jejuni infection in Colorado: identified risk factors. Am J Public Health.

[72] Conan A, O’Reilly CE, Ogola E, Ochieng JB, Blackstock AJ, Omore R (2017). Animal-related factors associated with moderate-to-severe diarrhea in children younger than five years in western Kenya: A matched case-control study. PLoS Negl Trop Dis.

[73] Gillespie I, O’Brien S, Penman C, Tompkins D, Cowden J, Humphrey T (2008). Demographic determinants for Campylobacter infection in England and Wales: implications for future epidemiological studies. Epidemiol Infect.

[74] Pyra M, Conover C, Howland J, Soyemi K (2012). Determinants of campylobacteriosis notifications in New Zealand. Epidemiol Infect.

